# Bee Habitat, but Not Bee Community Structure, Varies Across Grassland Management in Four National Parks in the Mid‐Atlantic, USA

**DOI:** 10.1002/ece3.70719

**Published:** 2024-12-17

**Authors:** Diane L. Larson, Michael Simanonok, Andrew Landsman, Jennifer L. Larson, Cora Davies, Clint R. V. Otto

**Affiliations:** ^1^ U.S. Geological Survey Northern Prairie Wildlife Research Center Jamestown North Dakota USA; ^2^ National Park Service Chesapeake & Ohio Canal National Historical Park – CHOH Williamsport Maryland USA; ^3^ U.S. Department of Agriculture Forest Service, Forest Health Protection St. Paul Minnesota USA

**Keywords:** floral diversity, forb, grassland management, habitat, native bees, pollinators

## Abstract

National Park Service units in the United States play a large role in providing habitat for native pollinators. In parks that are established to preserve cultural landscapes, park managers recognize an opportunity to improve pollinator habitat while maintaining historically accurate conditions. In this study, we document floral resources and native bees within managed park grasslands, with the goal of providing information to managers to help them maximize pollinator habitat while meeting other management objectives. The study was performed on 37 grassland properties in the mid‐Atlantic region of the eastern United States, distributed across four parks; each property was managed with one of three management types: cool‐season hayed, cool‐season pasture, or warm‐season meadows managed with multiple approaches. We surveyed bees and open flowers on 50‐m transects twice each year in 2021 and 2022. Repeated‐measures ANOVA models revealed that mean bee abundance, richness, evenness, and diversity did not vary among sites or management types. This finding was further supported by a principal coordinates analysis that showed that bee community composition was similar across management types. Nonetheless, we found evidence to indicate that the three management types did not produce equivalent habitat for bees. Species accumulation curves showed that the effective number of flower species was consistently lower in cool‐season pastures, relative to the other two management types. Furthermore, we detected positive correlations between bee and flower diversity metrics in one of the 2 years, indicating that floral metrics are associated with bee communities, at least under certain conditions. Collectively, our study suggests that cool‐season fields that are hayed and warm‐season meadows have higher floral diversity than cool‐season pastures within national park units of the mid‐Atlantic region, and this higher diversity of forbs has the potential to benefit native bee diversity.

## Graphical abstract

Park managers are interested in knowing the impacts of grassland management on bee communities and bee habitat. We found that forb diversity, but not bee community structure, varies across grassland management in four national parks in the Mid‐Atlantic, United States.

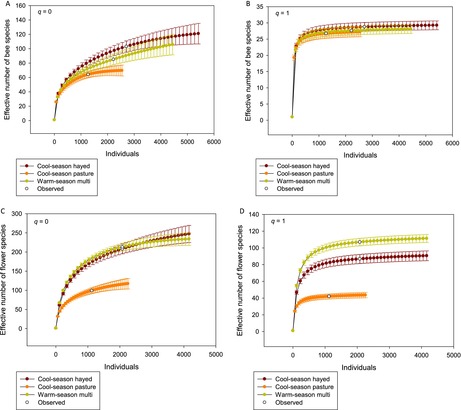

## 1 Introduction

Public lands can provide refugia for a variety of species in the face of foraging and nesting habitat loss to agriculture, urbanization, and other uses. The need for such refugia is especially strong for native bees, whose populations (Goulson 2019; Wagner 2020) and diversity (Zattara and Aizen 2021) have declined dramatically in recent years. Studies on US National Park Service (NPS) lands have documented extremely high bee species richness at Pinnacles National Park (Carril et al. 2018; Meiners, Griswold, and Carril 2019), the presence of rare and specialist species in a survey of 46 national park units across the United States (Rykken et al. 2014), and the importance of native bees in the pollination of a rare plant at Haleakala National Park (Krushelnycky 2014) and an endemic plant at Badlands National Park (Larson et al. 2014, 2021), among others. Nonetheless, with more than 32.4 million ha (80 million acres) of land protected by the NPS, there is a need to better understand the diversity and habitat requirements of native bee fauna residing therein. In particular, the NPS has recognized the need to understand the relationships between floral resources, which park resource managers can directly influence, and native bee communities on NPS lands to support pollinator populations and diversity more effectively.

Temperate grasslands are of conservation concern worldwide, with grassland conversion exceeding grassland protection by a ratio of 8:1 as of 2005 (Hoekstra et al. 2005). The mid‐Atlantic region of the eastern United States consists of small patches of grassland within a matrix of woodland, cropland, and developed areas. The majority of such grasslands are cultural in origin: prior to European settlement, patches of grassland were maintained by fires set by indigenous people. Afterward, livestock grazing and planting of forage grasses helped to maintain grassland habitat, albeit with potentially fewer forbs due to competition by the planted grasses. However, cessation of these disturbances facilitated woody plant encroachment and reduced grassland extent, thereby reducing available habitat for a number of grassland‐obligate and rare plant species (Tyndall 1992), and presumably, their pollinators.

In the administration of a national park, NPS managers must consider, maintain, and preserve cultural landscapes, so that visitors may experience cultural heritage in a physical form (54 U.S.C. 100101 *et seq*.). Particularly for parks established chiefly for their historic properties (Unrau and Williss 1987), the preservation of these landscapes and their characteristics and features is critical to convey to visitors the historical period of significance. Parks in the National Capital Region of the NPS, predominately those commemorating the Civil War, achieve these preservation mandates by creating and maintaining grasslands in historically open, agrarian landscapes through application of disturbances such as mowing, grazing, or burning to prevent woody species encroachment. Park managers often lease or rent grassland properties to cooperators who harvest hay or graze livestock on cool‐season grasses (e.g., planted agricultural grasses such as Timothy [ *Phleum pratense* ]). Some managers have seeded native warm‐season grasses and forbs, which are maintained by a variety of disturbances, including fire and mowing. Managers recognize that these disturbances also have the potential to create habitat for bees and other arthropod pollinators (McCullough, Angelella, and O'Rourke 2021), as well as declining grassland‐dependent birds (Massa et al. 2023). Because preservation and restoration of native biota is required by NPS management policies (2006) and various management approaches can be employed to maintain the historical landscape, managers can emphasize practices that produce the greatest benefits for native, grassland‐dependent animals.

The purpose of the current study is to document the floral resources and native bees that depend on them within NPS grasslands in the mid‐Atlantic region managed to portray the historical views and landscape configuration of the specific time period of cultural significance for each park. Our goal was to provide managers with information that will help them maximize pollinator habitat while concurrently meeting other management objectives. Specifically, we tested (1) whether bee abundance, richness, diversity, and community composition, as captured by bowl traps and hand netting, differed among grasslands managed as cool‐season hayfields, cool‐season pastures, or warm‐season meadows with multiple management approaches (hereafter, CSH, CSP, and WSM, respectively); (2) if floral density, richness, diversity, or community composition, measured on belt transects and botanist‐directed walks, varied among the three grassland management groups; and (3) if there was a relationship between floral and bee species abundance or diversity.

## 2 Methods

### 2.1 Study Area

**FIGURE 1:**
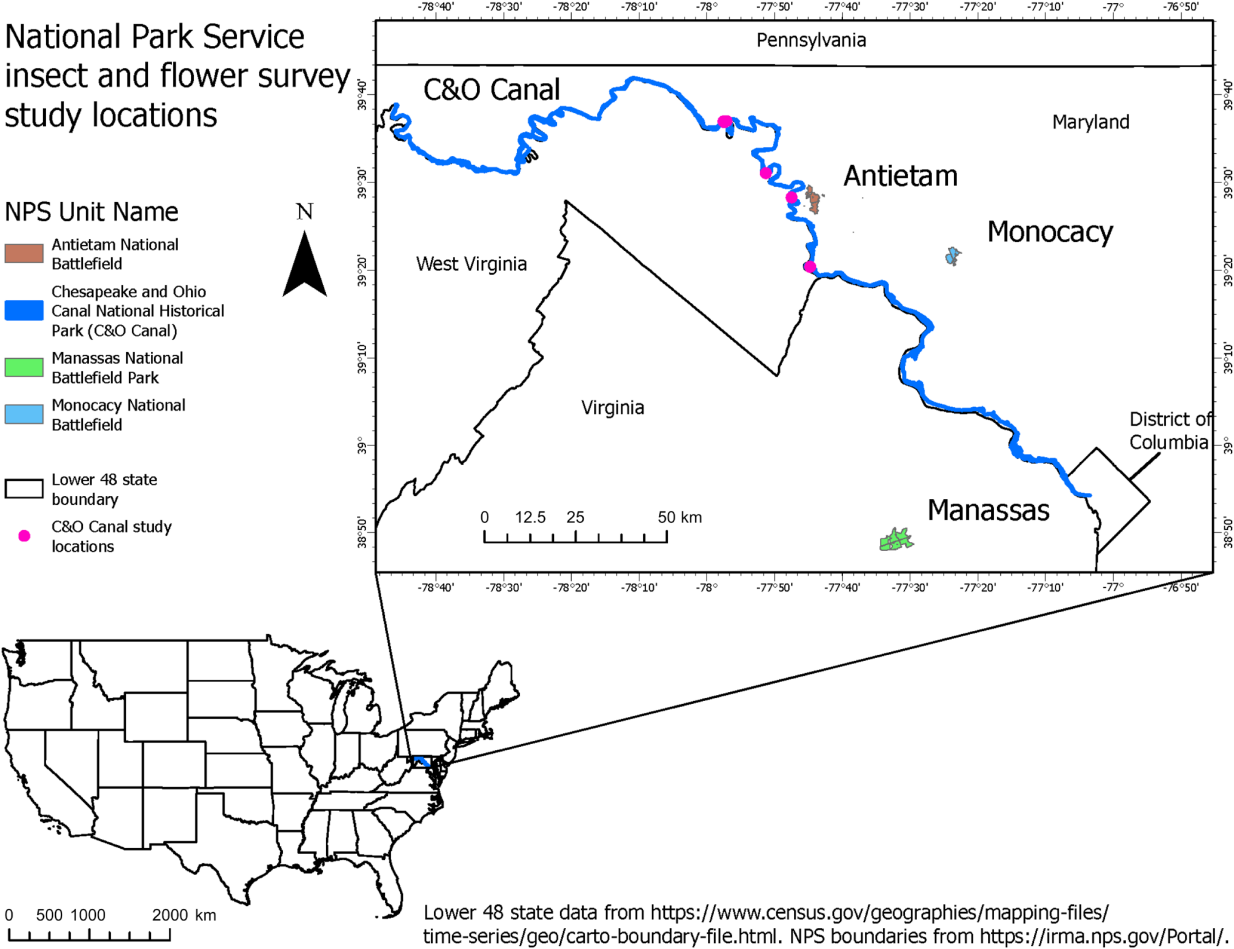
Map of four national park locations surveyed for floral resources and native bees in 2021 and 2022.

The study was carried out on 37 grassland properties in the mid‐Atlantic region of the eastern United States, distributed across four parks: Antietam National Battlefield (Antietam), Manassas National Battlefield Park (Manassas), Monocacy National Battlefield (Monocacy), and the Chesapeake and Ohio Canal National Historical Park (C&O Canal) (Figure 1). Each property was managed by one of the three management types (CSH, CSP, and WSM), but each of the three types was not present in all four parks (Table 1). Management for WSM consisted of prescribed fire, herbicide treatments, woody plant removal, and/or mowing. CSH fields were mowed and cut vegetation was baled and removed. CSP fields were grazed by cattle, but the stocking rate was not specified. Furthermore, frequency and intensity of management varied among and within parks, and the availability of management history likewise varied. Field sizes ranged from 1.2 to 33.7 ha (3–83 ac) with mean (±SE) field size of 8.3 ± 1.1 ha (20.5 ± 2.7 ac). Within each field, we randomly selected starting locations of 50 × 2 m transects. We scaled the number of transects per field with area: Fields < 4.04 ha (≤ 10 ac) contained two transects and one additional transect was added with every 2.02‐ha (5‐ac) increase in area up to a maximum of 10 transects per field. Whenever possible, we ensured sites were separated by > 1 km, and we achieved this for all sites in Antietam, Manassas, and C&O Canal. However, several sites at Monocacy were < 1 km apart due to the spatial arrangement of fields available for sampling. We included these close‐proximity sites in the analysis because they represented an ideal distribution of the different management types we aimed to investigate (Table 1).

**TABLE 1:** Number of fields within each management type for four national parks in the United States: Antietam National Battlefield, Chesapeake and Ohio (C&O) Canal National Historical Park, Manassas National Battlefield Park, and Monocacy National Battlefield.

Park	Cool‐season hayfields	Cool‐season pastures	Warm‐season meadows
Antietam	5	5	6
C&O Canal	5	—	—
Manassas	5	—	5
Monocacy	2	3	1

### 2.2 Bee Sampling and Floral Surveys

Bee sampling and floral surveys were conducted from May to September 2021 and 2022 using the same fields and transect locations each year. We used passive bowl sampling methods (Droege et al. 2016) at two randomly selected, 50‐m transects within each field, twice during each sample year (once in the first half and once in the second half of the season). Plastic 355‐mL (12 US fluid oz) white, blue, or yellow cups were placed on wire stands 0.3 m above the ground surface, every 5 m along the 50‐m transect, alternating colors. Bee bowls were filled with water and a drop of dish soap (Dawn Original Blue) to break surface tension and were collected after 24 h in place. Bowl samples exhibited well‐documented taxonomic biases (O'Connor et al. 2019; Portman, Bruninga‐Socolar, and Cariveau 2020), so we also conducted complementary hand netting of pollinating insects. For 30 min within each field, a technician hand netted all pollinating insects observed to be contacting the reproductive parts of flowers. Bees were placed in plastic vials and kept in coolers on ice until they could be transferred to a freezer in the lab. Bee sampling was only performed during clear, warm (≥ 16°C) days with < 30 km/h winds, from 08:00 to 17:00. Insect identification was performed by the Native Bee Inventory and Monitoring Lab of the US Geological Survey, Eastern Ecological Science Center.

We completed floral surveys at the same time as bee sampling by counting open flowers of each species, once in the first half of the season and once in the last half, on all of the 50 × 2 m transects described earlier. Prior to floral surveys, each plant species in a flower was photographed and a decision recorded about what would be considered the floral unit. For example, technicians recorded a sunflower inflorescence as one “floral unit” and a *Campanula* flower as one “floral unit”: Each would provide different rewards to visiting bees, but determining actual pollen and nectar rewards was beyond the scope of this project. Hereafter, floral units will be simply referred to as flowers. We used a small‐diameter white PVC pipe 2 m in length and marked at 25‐cm intervals for counting flowers within the 2‐m belt transects. Before counting open flowers along a transect, the surveyor scanned the site to assess relative abundance of flowering species. Extremely abundant flowers were counted within a smaller fraction of the belt transect (e.g., at 25‐cm width rather than 2 m) and later standardized to the full 2‐m width before analysis.

### 2.3 Data Analysis

A repeated‐measures ANOVA model was used to test for the effects of park, management, and year on the response variables (see below). Some parks did not contain fields of all management types; therefore, only the interaction term between park and management was included in the model (i.e., a means model). Year was also included in the model as a repeated measure, both as a main effect and in interaction with park × management. Field within park and management was included as a random effect. If the park × management × year term or the park × management term was significant, then contrast statements were used to test for differences among management types using only parks that had fields of both management types. We used PROC GLIMMIX in SAS (SAS Institute Inc., Cary, North Carolina, USA) to run the means model. Response variables were bee and flower abundance (i.e., mean number of bees captured in bowl traps or number of flowers counted on transects/field), and bee and flower species' richness, evenness, and Shannon's diversity, calculated using the Summary function in PC‐ORD version 7.08 (McCune and Mefford 2018). To avoid confounding field size with species richness, we used the two transects where bee bowls were deployed for both bee and floral metrics.

To test how bee community composition responded to grassland treatment, we used a principal coordinates analysis (PCoA, function dbrda in vegan 2.6‐8, Oksanen et al. 2022) of the bee communities present at each field in each sampling year. We then performed a pairwise multiple comparison test with 999 permutations that average ordination scores among factors (function pairwise.factorfit in RVAideMemoire, Herve 2023) to differentiate among grassland treatments; furthermore, to account for potential differences among park management practices, we included park (Antietam, C&O, Manassas, or Monocacy) as “strata” in the model, which limits the permutations among treatments to within each park. We repeated these analyses with floral community composition data collected on transects to assess floral community responses to grassland treatments.

We used the iNext package (Hsieh, Ma, and Chao 2016) in R 4.2.1 (R Core Team 2022) to calculate Hill diversity (effective number of species; Chao and Jost 2015) at *q* = 0 and 1 as a function of number of individuals observed in each management type. *q* represents sensitivity to species relative abundance, which is greatest at *q* = 0 and declines as *q* increases: *q* = 0 corresponds to species richness and *q* = 1 to Shannon diversity (Chao and Jost 2015). We used the sum of individual bees captured in bowls and nets or individual flowers counted on transects rather than the number of transects as our measurement of effort due to different sampling efforts across the three management types. This allowed us to use all the data collected on transects. We used the function SimilarityMulti in SpadeR to estimate the Horn size–weighted similarity (Chao et al. 2016) in bee and flower species among the three management types.

To test for a relationship between floral and bee abundance and diversity, we used bee and flower abundance, species richness, Shannon's diversity, and evenness from the Summary function in PC‐ORD as described earlier. We analyzed years separately because both bees and flowers are subject to year‐to‐year variation. Because the relationship between bees caught in bowl traps and the habitat is generally weak, with captures in bowls declining as flower abundance increases (Baum and Wallen 2011; Larson et al. 2024; Pei et al. 2022), we used only bees captured in nets for this analysis. Correlation coefficients were calculated with proc. corr in SAS version 9.4.

## 3 Results

### 3.1 Bee and Flower Abundance and Diversity

**FIGURE 2:**
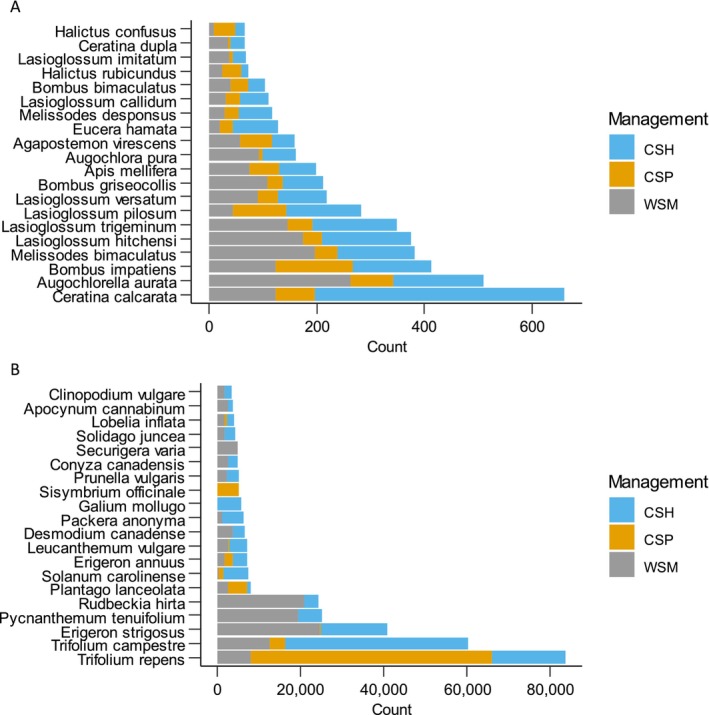
Counts of 20 most common bee (A) and flower (B) species observed at 37 National Park Service units in the United States in 2021 and 2022. Grassland management type: Cool‐season hayfields (CSH), cool‐season pastures (CSP), and warm‐season meadows (WSM).

We collected 6199 individual bees (3489 in 2021 and 2710 in 2022) of 128 taxa (including eight species groups; Table S1, Figure 2A). More than 60% of these bee species were captured < 10 times over the 2 years of the study and 43 species were singletons or doubletons. The 24 most common species accounted for > 86% of all captures and they were all in either the Apidae (including 199 *Apis mellifera* ) or Halictidae (Table S2, Figure 2A). We counted 412,518 flowers (187,571 in 2021 and 224,947 in 2022) of 287 taxa (Table S2, Figure 2B). Fifty‐three percent of the floral species were native to the study area (Table S3).

Bee relative abundance (*F*_1,28_ = 13.94, *p* < 0.001) and richness (*F*_1,28_ = 5.48, *p* = 0.027) were higher in 2021 than those in 2022, but evenness was higher (*F*_1,28_ = 4.50, *p* = 0.043) in 2022 than that in 2021 (Table 2). Although the overall test for significance of the park × management interaction on bee species richness was significant (*F*_8,28_ = 3.25, *p* = 0.001), none of the contrasts of interest were significantly different.

**TABLE 2:** Mean and standard error by year for bee and flower abundance and diversity metrics for 37 National Park Service units in the United States in 2021 and 2022. Bold *p*‐values indicate significant differences (*p* < 0.05) between years.

	Variable	2021	2022	*F*_1,28_	*p*
Bees	Abundance	1.91 (0.16)	1.17 (0.16)	13.94	**< 0.001**
	Richness	19.36 (0.87)	16.49 (0.87)	5.48	**0.027**
	Evenness	0.81 (0.016)	0.85 (0.016)	4.5	**0.043**
	Shannon diversity	2.37 (0.059)	2.32 (0.059)	0.3	0.600
Flowers	Abundance	0.50 (0.068)	0.62 (0.068)	10.08	**0.004**
	Richness	27.11 (1.85)	31.90 (1.85)	13.28	**0.001**
	Evenness	0.94 (0.005)	0.93 (0.005)	0.04	0.840
	Shannon diversity	3.03 (0.066)	3.16 (0.066)	8.06	**0.008**

In contrast to bees, flower relative abundance (*F*_1,28_ = 10.08, *p* = 0.004), richness (*F*_1,28_ = 13.28, *p* = 0.001), and Shannon diversity (*F*_1,28_ = 8.06, *p* = 0.008) were all lower in 2021 than those in 2022 (Table 2). Despite an overall significant effect of park × management × year for flower abundance (*F*_8,28_ = 2.66, *p* = 0.026), none of the contrasts of interest were significantly different. The overall test of park × management × year for floral species evenness was significant (*F*_8,28_ = 2.82, *p* = 0.020): CSH exhibited greater evenness than CSP in 2021 and WSM had greater evenness than CSP in both years (Table 3).

**TABLE 3:** Results of planned contrasts between flower evenness estimates for park × management type × year based on data from 37 National Park Service units in the United States in 2021 and 2022. CSH, cool‐season hayed; CSP, cool‐season pasture; WSM, warm‐season multiple management types. Contrasts in bold indicate management types that differed significantly (*p* < 0.05) within the year specified.

Contrast	Management	Year	Mean	SE
**CSH vs. CSP, 2021**	CSH	2021	0.935	0.011
	CSP	2021	0.904	0.010
CSH vs. CSP, 2022	CSH	2022	0.921	0.011
	CSP	2022	0.915	0.010
CSH vs. WSM, 2021	CSH	2021	0.935	0.011
	WSM	2021	0.950	0.008
CSH vs. WSM, 2022	CSH	2022	0.921	0.011
	WSM	2022	0.951	0.014
**CSP vs. WSM 2021**	CSP	2021	0.904	0.010
	WSM	2021	0.953	0.014
**CSP vs. WSM 2022**	CSP	2022	0.915	0.010
	WSM	2022	0.951	0.014

### 3.2 Bee and Floral Community Composition

**FIGURE 3:**
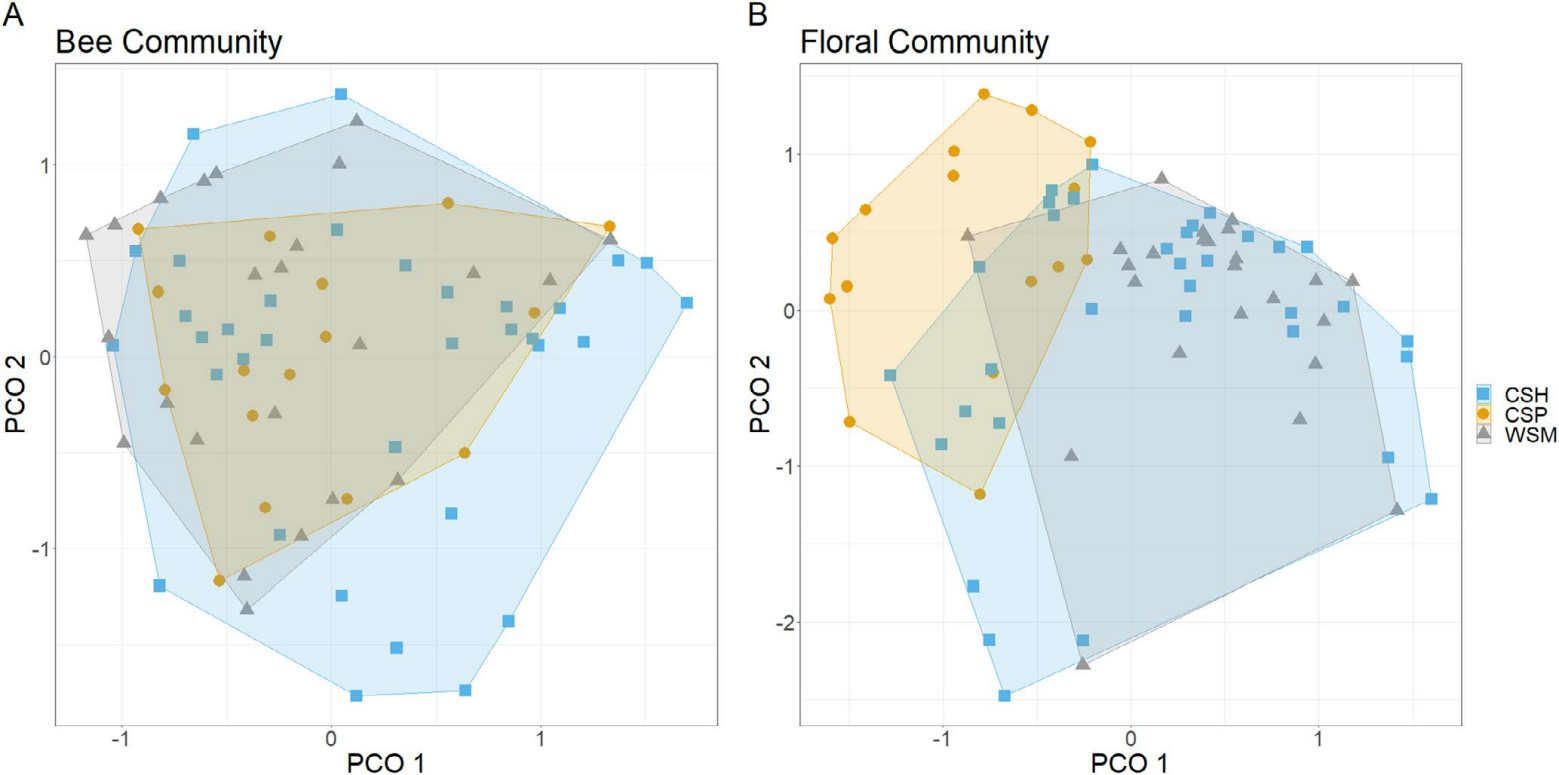
Principal coordinates analysis of bee (A) and floral (B) communities at each study field (*n* = 37) in each year, shaded by grassland treatment type: Cool‐season hayfields (CSH), cool‐season pastures (CSP), and warm‐season meadows (WSM) at 37 National Park Service units in the United States in 2021 and 2022.

Bee community composition was similar among treatments (CSP vs. CSH *p* = 0.590, CSH vs. WSM *p* = 0.110, CSP vs. WSM *p* = 0.590, Figure 3A) and did not vary between sampling years (*p* = 0.190). CSP had compositionally different floral communities from both CSH and WSM (*p* < 0.010, Figure 3B), but CSH and WSM were compositionally similar (*p* = 0.210, Figure 3B). Floral community composition did not vary between sampling years (*p* = 0.710). As a percentage of counted flowers over the 2 years, WSM had more native and fewer introduced species and CSP had the most introduced species and fewest natives (Table S3).

### 3.3 Relationship Between Diversity and Management Types

**FIGURE 4:**
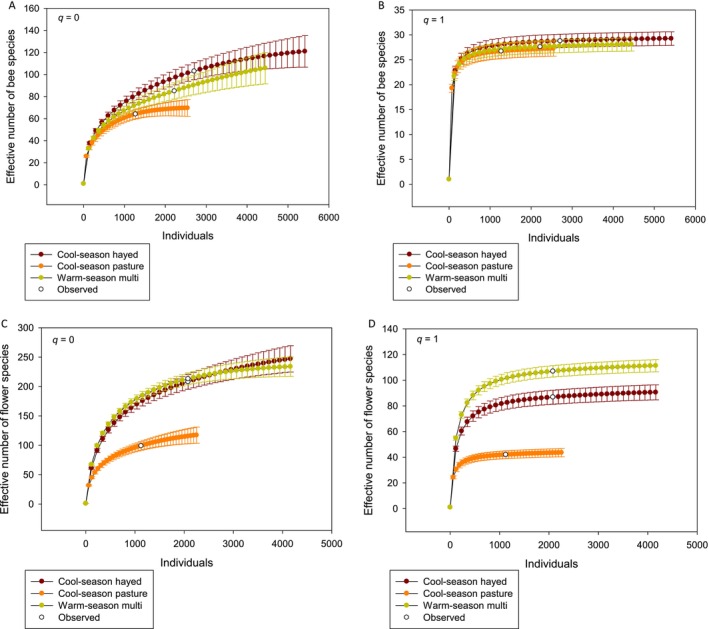
Effective number of bee species at *q* = 0 (A) and *q* = 1 (B), and effective number of flower species at *q* = 0 (C) and *q* = 1 (D) as a function of number of individuals observed at 37 National Park Service units in the United States in 2021 and 2022 were pooled. Values above the observed number of species are extrapolated and those below are rarefied.

Abundance‐weighted bee and flower species richness (i.e., *q* = 0) showed similar patterns among management types: effective numbers of both bee and flower species were lower in CSP than those in CSH‐ or WSM‐managed fields (Figure 4A,C). Conversely, Shannon diversity (i.e., *q* = 1) was similar for bees in all three management types (Figure 4B); however, WSM contained the highest Shannon diversity of flowers and CSP contained the lowest (Figure 4D). Estimated mean Horn size‐weighted bee abundance similarity at *q* = 1 was 0.91 (SE = 0.0059), indicating considerable similarity among bee species within the three management types. For flowers, estimated mean Horn size‐weighted species abundance similarity at *q* = 1 was 0.79 (SE = 0.013), consistent with the greater differences shown in Figure 4D.

### 3.4 Relationship Between Floral Resources and Netted Bee Species Diversity

**FIGURE 5:**
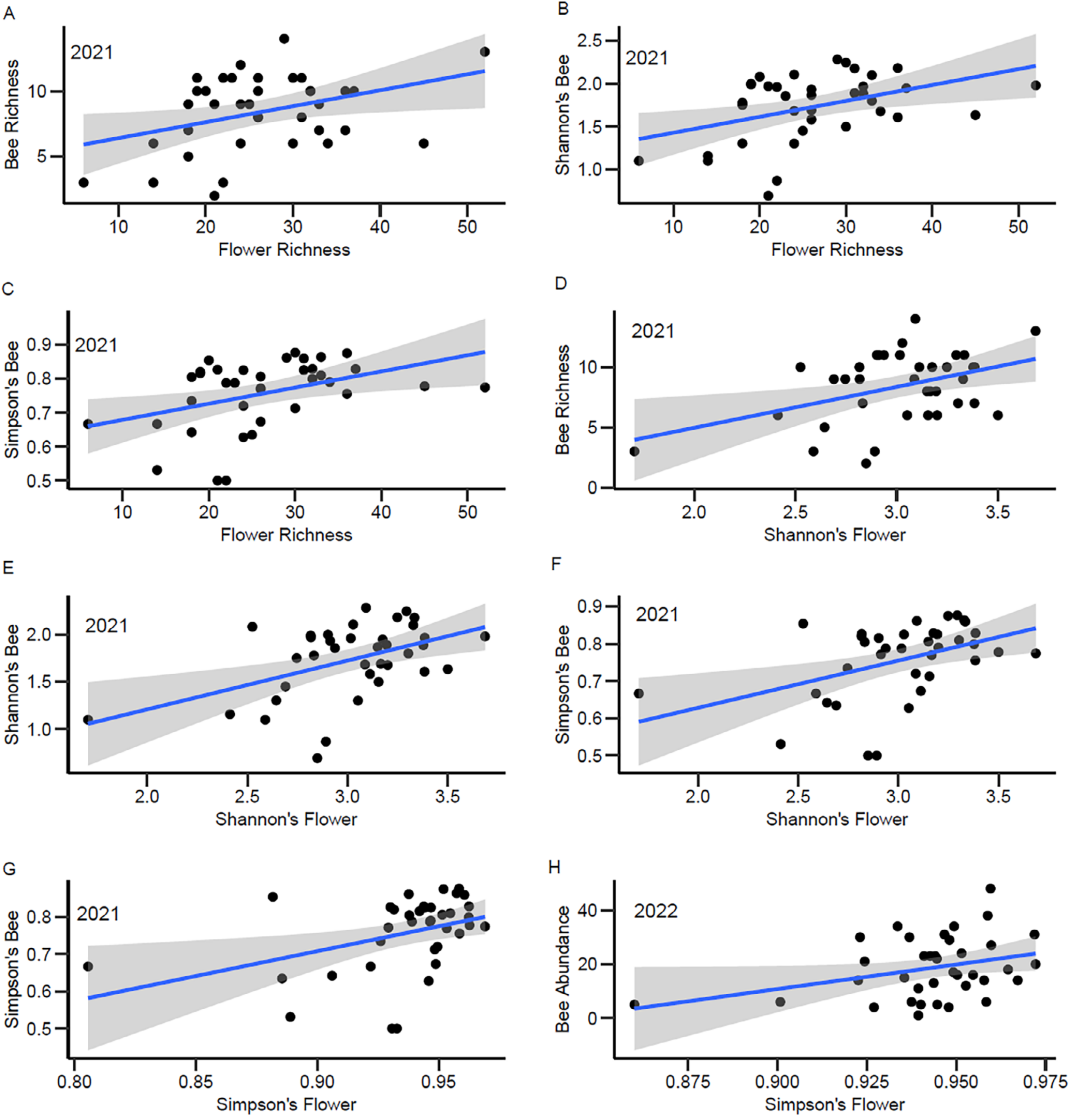
Significant correlations (refer to Tables 4 and 5) between flower richness and bee richness (A), Shannon's Bee Diversity Index (B), Simpson's Bee Diversity Index (C), Shannon's Flower Diversity Index and bee richness (D), Shannon's Bee Diversity Index (E), Simpson's Bee Diversity Index (F), Simpson's Flower Diversity Index and Simpson's Bee Diversity Index (G), and Simpson's Flower Diversity Index and bee mean abundance (H). Year is displayed in the upper corner left corner of each graph. For simplicity, only significant correlations from Tables 4 and 5 are displayed. Data were collected at 37 National Park Service units in the United States in 2021 and 2022. The shaded areas represent 95% confidence intervals.

Flower richness was positively correlated with bee richness, Shannon diversity, and Simpson's diversity in 2021 (Table 4; Figure 5), but these relationships were not significant in 2022 (Table 5). In addition, both indices of flower diversity were positively correlated with bee richness, Shannon diversity, and Simpson's diversity in 2021. We observed no correlation between flower evenness and any metric related to bee richness or diversity. In 2022, the only significant correlation we detected was between Simpson's diversity index for flowers and bee abundance (Table 5; Figure 5). In all cases, these correlations were relatively weak, with *R* ^2^ < 0.30 for all comparisons (Tables 4 and 5; Figure 5). We had only one site with low flower richness (< 10 species) and diversity, which was in part responsible for some of the correlations we detected. Removal of this site from the analysis still resulted in significant correlations for five of the eight correlations observed in Tables 4 and 5.

**TABLE 4:** Pearson correlation coefficients and their significance (*P*) between bee and flower diversity metrics based on data from 37 National Park Service units in the United States in 2021. Significant correlations (*p* < 0.05) are in bold. For all correlations, *n* = 37.

Flower	Bee				
	Total^a^	Richness	Evenness	Shannon's	Simpson's
Total^b^	0.21	0.19	0.056	0.24	0.25
*p*	0.21	0.25	0.74	0.15	0.13
Richness	0.32	**0.37**	0.011	**0.42**	**0.41**
*p*	0.053	**0.023**	0.95	**0.009**	**0.011**
Evenness	−0.23	−0.032	0.18	0.026	0.087
*p*	0.17	0.853	0.30	0.887	0.61
Shannon's	0.30	**0.42**	−0.022	**0.48**	**0.45**
*p*	0.075	**0.009**	0.91	**0.003**	**0.005**
Simpson's	0.20	**0.38**	−0.012	**0.43**	**0.40**
*p*	0.23	**0.021**	0.94	**0.007**	**0.013**

^a^ Number of bees netted.

^b^ Sum of flowers observed.

**TABLE 5:** Pearson correlation coefficients, their significance (*p*), and sample size (*n*) between bee and flower diversity metrics based on data from 37 National Park Service units in the United States in 2022. Significant correlations (*p* < 0.05) are in bold.

Flower	Bee				
	Total^a^	Richness	Evenness	Shannon's	Simpson's
Total^b^	0.16	0.18	−0.24	0.19	0.16
*p*	0.33	0.28	0.16	0.26	0.36
*n*	37	37	36	37	37
Richness	0.25	0.26	−0.10	0.27	0.24
*p*	0.13	0.12	0.55	0.10	0.16
*n*	37	37	36	37	37
Evenness	−0.086	−0.075	0.31	−0.054	−0.018
*p*	0.61	0.66	0.07	0.75	0.92
*n*	37	37	36	37	37
Shannon's	0.32	0.31	−0.10	0.30	0.23
*p*	0.052	0.066	0.57	0.076	0.17
*n*	37	37	36	37	37
Simpson's	**0.33**	0.30	−0.12	0.27	0.18
*p*	**0.047**	0.070	0.48	0.11	0.28
*n*	**37**	37	36	37	37

^a^ Number of bees netted.

^b^ Sum of flowers observed.

## 4 Discussion

The overarching goal of this study was to provide NPS managers with information to optimize grassland habitat for native bee pollinators while maintaining esthetic landscapes consistent with each park's historical context. Although bee community composition was remarkably similar across the 37 sampled sites in our study and mean bee abundance, richness, evenness, and diversity did not vary among sites or management types, we found evidence to indicate that the three management types did not produce equivalent habitat for bees. In particular, floral evenness was consistently lower in CSP, and the effective numbers of bee and flower species were also lower in CSP at *q* = 0 (and for flowers also at *q* = 1). Significant positive correlations between bee and flower diversity metrics in one of the 2 years suggest that these floral metrics can influence bee communities, at least under certain conditions. This study represents one of the largest samplings of bees on federal lands in the Mid‐Atlantic and helps build an inventory regarding the diversity and distribution of these species while providing necessary information for land managers in the region.

The similarity we observed in bee community composition across the study area is not unexpected. The landscape we studied had been significantly altered during European colonization as extensive forests were cleared to make way for crops and livestock pasture (Peterjohn and Sauer 1999). This alteration would have created a strong ecological filter (Mayfield et al. 2005). The only bee species that would have persisted through this change and subsequent reforestation as fields were abandoned were either those sufficiently generalized in their habitat requirements or those that were able to expand their range from the previously existing grassland patches. Similarly, Hung et al. (2019) compared taxonomic and functional attributes of bee assemblages in reserves with those in fragmented scrub habitat in California and found that bees inhabiting fragmented habitats were generalists that could persist in a wide array of ecological contexts. Doré, Fontaine, and Thébault (2021), in an analysis of a worldwide dataset of 295 pollination networks, likewise found that anthropogenic pressure (measured as the Human Influence Index) increased generalism in the network structure, even in the absence of effects on species richness. Other works have consistently shown similar effects of human‐caused landscape modification to bee communities (e.g., Harrison, Gibbs, and Winfree 2017).

Despite the overall similarity of bee communities across the study area, bee species abundance‐based accumulation curves indicated fewer effective number of species in fields managed as CSP than in those managed as either CSH or WSM at *q* = 0, that is, when rare species are weighted equivalently to common, as in species richness (Roswell, Dushoff, and Winfree 2021). In contrast, bee species abundance‐based accumulation curves for the three management types were nearly identical at *q* = 1, that is, when common species were more heavily weighted, as in Shannon diversity (Roswell, Dushoff, and Winfree 2021). Together, these results indicate that it is the presence of rare species that separates bee communities in CSH and WSM from those in CSP. Most of the species we documented were rarely collected: Approximately 60% of species were represented by 10 or fewer individuals. Conversely, just 17 species comprised approximately 80% of all individual bees collected. These commonly collected halictid and apid species were known to be common across their wide distribution in the eastern United States (Mitchell 1960, 1962). As others have noted, common bee species are present in most samples (Grundel et al. 2011), as we found at *q* = 1.

Turning to flower diversity, at *q* = 0, the abundance‐based species accumulation pattern in management types is similar between flowers and bees, but at *q* = 1, the three management types further separate, so that WSM floral diversity asymptotes at a higher effective number of species than CSH, which is higher than CSP. Differences in floral diversity among the three management types therefore reside in both rare and common species. The correlations we observed in 2021 suggest that bee species diversity can track floral diversity, as many others have also reported (Potts et al. 2003; Lane et al. 2020; Theodorou et al. 2020; Kuhlman et al. 2021). This suggests that, despite the lack of observable differences in bee communities across management types, WSM sites have the capacity to support a more species‐rich bee community than do other management types because of their greater floral diversity. The reason for the differences in floral diversity may be related to the initial management objectives for the fields. The CSH and CSP fields would have been optimized for livestock forage value, thus emphasizing the grass component, whereas WSM fields were more purposefully planted for the benefit of pollinators and other grassland‐dependent wildlife.

The weak correlation between bee abundance and floral Simpson's diversity, and the absence of any other significant correlations in 2022, point to a potential effect of the often observed year‐to‐year variation in bee populations (Roubik 2001; Tepedino and Parker 2022). The 22% fewer bees captured in 2022 may not have been adequate to detect broad‐scale correlations such as we measured in 2021. (Conversely, the 17% greater floral resource abundance in 2022 may also have weakened any potential correlation.) The floral evenness component of diversity also varied among the management types, again with CSP lagging CSH and WSM in flower evenness. Although generally understudied, we note that others have found a positive relationship between bloom evenness and pollinator diversity (Braatz et al. 2021) as well as a negative association between evenness and ecosystem function, as measured by the seed set (Stavert et al. 2019). Despite the variation we found in floral evenness among management types, we did not observe any correlation between floral evenness and bee diversity or abundance. It is important to note that in this study, evenness refers only to open flowers, so it ignores the abundance of cool‐season grasses that were planted in both CSH and CSP, which could be expected to dominate the vegetation in both management approaches. In contrasting CSH and CSP, haying does not discriminate among plant species as grazers would be expected to do; spiny amaranth ( *Amaranthus spinosus* ) and some species in the Brassicaceae that were more frequently encountered in pastures were likely unpalatable to cattle. Moreover, the larger proportion of native flowers in WSM would be expected to support more native bees, whereas the introduced flower species that dominated CSP may attract more non‐native bees, such as honeybees ( *A. mellifera* ) (Simanonok, Otto, and Buhl 2021).

A comparison of mean metrics for both bees and flowers, as shown by the results of our means model, indicated little variation with which to make conclusions about the effect of management types on bee diversity. Nonetheless, the abundance‐based species accumulation curves showed clear differences among management types with respect to both the effective number of bee and flower species, with CSP consistently supporting the fewest species. Because the effective number of species expresses diversity as the number of equally abundant species (Chao, Chiu, and Jost 2014), it allows a valid comparison among sites with different species' abundance curves (Chao, Gotelli, et al. 2014). Our study was not designed to determine mechanisms driving differences in diversity among management types, but we note that grazing can be associated with increased soil bulk density (Larson et al. 2020), which may alter nesting site availability for ground‐nesting bees. In addition, herbivores may selectively graze particular plant species and thereby change plant community composition or flower production (DeBano et al. 2016; Li et al. 2021), whereas fire has been found to lengthen the flowering period in California grasslands (Mola and Williams 2018). We lack detailed information on timing and intensity of the different management approaches, but these can have strong effects on the outcome. For example, Lazaro et al. (2016) found a unimodal distribution for pollinator abundance and richness as grazing intensity increased on the Greek island of Lesvos, although there was some seasonal variation in this result. Understanding the mechanisms driving difference in diversity among management types would require greater control, standardization, and recording of management actions and timing of management than what was available in our study. Ideally, managers and researchers would identify management actions of interest, such as prescribed fire, and implement a before–after–control–impact (i.e., BACI) design to understand how bee and plant communities respond. This more structured design would allow managers to identify management targets, such as frequency of burning, and how it impacts pollinator communities and the floral resources on which they rely.

Consideration could also be given to the effects of meadow management strategies on other taxa, including grassland birds, which can be more species rich and more abundant in WSM (Giuliano and Daves 2002). Strategies employed by managers to maintain WSM, such as prescribed burning, have also been correlated with greater bee richness and abundance (Mason et al. 2021). Given these considerations, managing as CSH or WSM may improve floral and bee diversity over CSP.

## Author Contributions

**Diane L. Larson:** conceptualization (equal), data curation (supporting), formal analysis (lead), funding acquisition (equal), methodology (equal), visualization (equal), writing – original draft (lead), writing – review and editing (equal). **Michael Simanonok:** conceptualization (equal), formal analysis (equal), investigation (lead), methodology (supporting), project administration (equal), supervision (equal), writing – original draft (supporting), writing – review and editing (supporting). **Andrew Landsman:** formal analysis (supporting), methodology (supporting), writing – review and editing (equal). **Jennifer L. Larson:** investigation (equal), methodology (equal), visualization (lead), writing – review and editing (supporting). **Cora Davies:** investigation (supporting), project administration (supporting), writing – review and editing (supporting). **Clint R. V. Otto:** conceptualization (lead), data curation (lead), funding acquisition (equal), methodology (supporting), project administration (supporting), writing – review and editing (equal).

## Conflicts of Interest

The authors declare no conflicts of interest.

## Supporting information


Table S1

## Data Availability

Data supporting this study are publicly available as a US Geological Survey data release (Otto and Larson 2024).
